# Extra-urogenital infection by *Mycoplasma hominis* in transplant patients: two case reports and literature review

**DOI:** 10.1186/s12879-023-08593-2

**Published:** 2023-09-14

**Authors:** Afrinash Ahamad, Fainareti N. Zervou, Maria E. Aguero-Rosenfeld

**Affiliations:** 1 Clinical Laboratory Sciences Program, School of Health Profession, Stony Brook University, Stony Brook, NY USA; 2 Department of Neuroscience and Behavior, Stony Brook University, Stony Brook, NY USA; 3 Department of Pathology, Clinical Microbiology Laboratory, NYU Langone Health, New York, NY USA; 4 Department of Medicine, NYU Langone Health, New York, NY USA

**Keywords:** *Mycoplasma hominis*, Extragenital infection, Solid organ transplant patients, Beta-lactam resistance, Heart and lung transplant, Thioglycolate broth, Diagnostic microbiology laboratory

## Abstract

**Background:**

*Mycoplasma hominis* is a facultative anaerobic bacterium commonly present in the urogenital tract. In recent years, *M. hominis* has increasingly been associated with extra-urogenital tract infections, particularly in immunosuppressed patients. Detecting *M. hominis* in a diagnostic laboratory can be challenging due to its slow growth rate, absence of a cell wall, and the requirements of specialized media and conditions for optimal growth. Consequently, it is necessary to establish guidelines for the detection of this microorganism and to request the appropriate microbiological work-up of immunosuppressed patients.

**Case Presentation:**

We hereby present two cases of solid organ transplant patients who developed *M. hominis* infection. Microscopic examination of the bronchial lavage and pleural fluid showed no microorganisms. However, upon inoculating the specimens onto routine microbiology media, the organism was successfully identified and confirmation was performed using 16S rDNA sequencing. Both patients received appropriate treatment resulting in the resolution of *M. hominis* infection.

**Conclusions:**

The prompt detection of *M. hominis* in a clinical specimen can have a significant impact on patient care by allowing for early intervention and ultimately resulting in more favorable clinical outcomes, especially in transplant patients.

## Introduction

*Mycoplasma species* belong to the Mollicutes class and *Mycoplasmataceae* family [1], commonly found in humans and mammals [2]. *Mycoplasma hominis* inhabits the lower genitourinary tract of sexually active men and women [3]. Extragenital presence of *Mycoplasma hominis* is relatively rare, and it is believed that the host’s immune status may be a predisposing factor [4]. The organism’s ability to transition from a commensal to a pathogenic state underscores the need for a more comprehensive understanding of the variables that contribute to the onset of infection [5]. The identification of *Mycoplasmas* using traditional techniques such as microscopy and culture in clinical microbiology poses a challenge primarily due to the absence of a cell wall [6]. Timely identification of *M. hominis* is crucial for appropriate treatment. Vulnerable patients, such as solid organ transplant recipients, are at risk of invasive infection, as the most empiric antibiotic regimen targets the cell wall and *M. hominis* lacks it. We herein present two cases of patients with heart and bilateral lung transplants who developed *M. hominis* infection and the organism was detected using conventional microbiological methods. Also, we have summarized the cases of *M. hominis* in heart and lung transplant patients and provided current diagnostic practices.

### Case 1

**Fig. 1:**
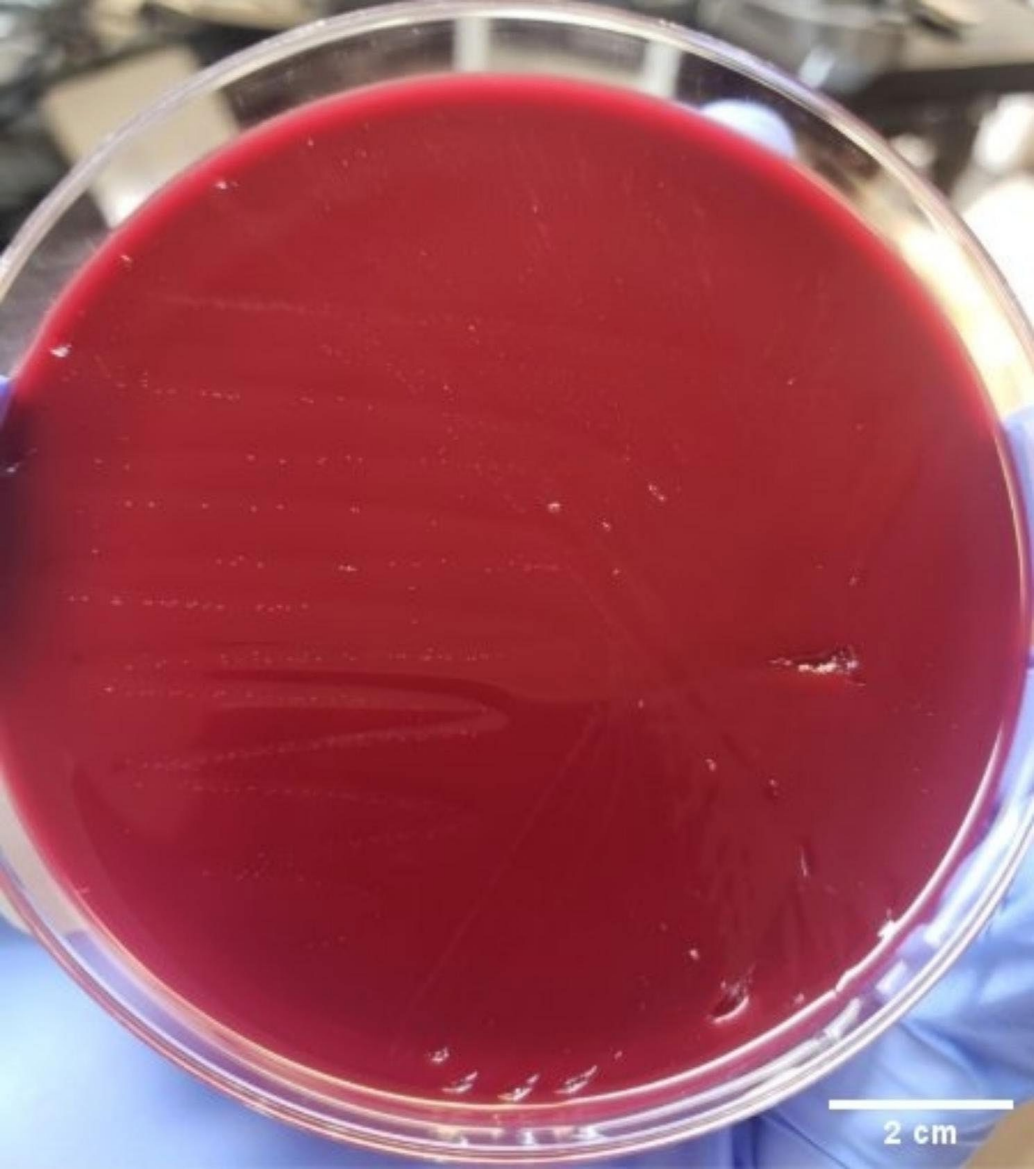
Shows pinpoint, flat, translucent colonies of *Mycoplasma hominis* isolated on blood agar following 48 h of incubation at 35^o^C in 5% CO_2_ atmosphere. Scale = 2.0 cm

A 58-year-old male with a history of idiopathic pulmonary fibrosis and significant peripheral vascular disease was admitted to the hospital for bilateral lung transplantation. The patient received basiliximab for induction suppression followed by tacrolimus, mycophenolate mofetil, and prednisone for immunosuppression maintenance. The donor was a male in his thirties who died of a drug overdose. The donor’s chest X-ray revealed left lower lung pneumonia, and bronchial lavage culture was positive for *Klebsiella pneumoniae*, therefore, the recipient was administered cefepime. Complete blood count (CBC) result from 7 days post-transplant showed leukocytosis (15.4 × 10^9^ cells/L [reference range 4-10 × 10^9^ cells/L]) and thrombocytosis (448 × 10^3^/µL [reference range 150–400 10^3^/µL]). A nasopharyngeal swab submitted for SARS CoV-2 PCR performed on the Cepheid Xpert Xpress was negative. Blood cultures were collected in addition to sputum samples and sent to clinical microbiology laboratory for culture. After 5 days of incubation in the BacT/ALERT 3D Microbial Identification System, blood cultures were reported negative. The sputum Gram stain and culture reported normal oropharyngeal flora. The patient’s chest X-ray showed significant bibasilar atelectasis worst on the right despite an aggressive airway clearance regimen, and worsened right pleural effusion. The patient underwent bronchoscopy, and the bronchoalveolar lavage (BAL) and right pleural fluid were sent to the microbiology laboratory for culture. Bronchial lavage and cyto-centrifuged pleural fluid Gram stain showed many WBCs and no organism. Pleural fluid inoculated in thioglycolate broth (Becton Dickinson BBL) and solid media, Blood (BD BBL), Chocolate (BD BBL) and MacConkey (BD BBL) agar were incubated at 35 ^o^C. After 48 h of incubation, slightly hazy thioglycolate broth was centrifuged, and the pellet was inoculated on blood agar and incubated at 35 ^o^C in 5% CO_2_. After 48 h of incubation, pinpoint colonies appeared on the Blood agar (BD BBL) (Fig. 1), and the initial plates inoculated with the specimen remained negative for the organism. *M. hominis* was identified using VITEK MS Matrix- Assisted Laser Desorption/Ionization Time-of-Flight (MALDI-TOF) (BioMérieux, USA), followed by confirmation using 16S rDNA sequencing (ARUP, Salt Lake City, Utah). The patient was administered with 200 mg of doxycycline once, followed by 100 mg q12h for 14 days, resulting in a remarkable resolution of leukocytosis from 15.4 × 10^9^ cells/L at the onset of therapy to 7.1 × 10^9^ cells/L at the completion of therapy. The pleural effusion was drained, and the subsequent imaging showed resolution of the right pleural effusion.

### Case 2

A 21-year-old male with a history of Becker Muscular Dystrophy (BMD) and cardiomyopathy, and who underwent orthotopic heart transplant (OHT), presented with multiple episodes of allograft rejection. He was subsequently admitted to the hospital 614 days after the transplant, following a brief period of cough and upper respiratory tract symptoms. He was on tacrolimus, mycophenolate mofetil, belatacept and prednisone for maintenance immunosuppression. A nasopharyngeal swab was positive for Rhinovirus/Enterovirus using the BioFire Respiratory panel and negative for SARS CoV-2 PCR performed on the Cepheid Xpert Xpress. The patient had leukocytosis (15.7 × 10^9^ cells/L [reference range 4-10 × 10^9^ cells/L]). The patient had worsening cardiac function requiring implantation of a total artificial heart (TAH) and extracorporeal membrane oxygenation (ECMO). The patient developed purulent respiratory secretions at around 2 weeks of hospitalization, and right basilar consolidation. Right bronchial lavage was collected and sent to clinical microbiology laboratory for culture. The Gram stain of cyto-centrifuged bronchial lavage showed innumerable WBCs, rare epithelial cells, and no organism. The specimen was inoculated on Blood (BD BBL), Chocolate (BD BBL) and MacConkey (BD BBL) agar, and after 48 h of incubation at 35 ^o^C small pinpoint colonies appeared on the blood agar. The specimen showed > 100,000 CFU/ml *Mycoplasma hominis* confirmed by 16S rDNA sequencing (ARUP, Salt Lake City, Utah). No other organism was identified on bacterial, fungal, and acid-fast bacilli cultures and multiplex PCR assay panels. The patient was treated with minocycline 200 mg once followed by 100 mg q12h for 14 days, and renally adjusted levofloxacin equivalent to 750 mg daily. There was an improvement in secretions, and subsequent BAL cultures were negative for *M. hominis.* The patient’s hospital course was further complicated by vasculitis, ischemic colitis with peritonitis, and disseminated histoplasmosis and he transitioned to hospice care two months after treatment for *M. hominis* infection.

## Discussion

*Mycoplasma hominis* is a facultative urogenital commensal microorganism reported in 29% of men and 75% of women. It causes pelvic inflammatory disease (PID), urinary tract infection (UTI), urethritis, and cervicitis [7, 8, 3]. The microorganism can also cause extragenital infections, such as pneumonia, septic arthritis, and intracranial subdural empyema [9–11]. In addition, *M*. *homini*s has been reported in 8% of patients with chronic respiratory disease, and is also detected in the respiratory secretions of 1–3% of healthy individuals [12]. The presence of *M. hominis* in transplant recipients can be donor-driven [13], however, in our cases, the exact source of infection in both patients remains unknown.

*Mycoplasmas* are classified as Mollicutes (Latin) meaning “soft skin” referring to the absence of peptidoglycan cell wall and their inability to be stained with the Gram stain [1]. The membrane of *M. hominis* is simple, composed of fatty acids, cholesterol, and complex lipids. Due to limited biosynthetic capabilities, *M. hominis* survival depends on the factors produced by the host. The organism is slow growing, and appears as tiny pinpoint colonies on the routinely used media such as blood agar [14, 15]. *M. hominis* has a unique metabolism requirement. It is a non-glycolytic organism, lacks genes encoding for the pyruvate dehydrogenase complex, and energy production is independent of the oxidation of pyruvate to acetyl-CoA. The presence of the gene acetate kinase (*ackA*) in *M. hominis* suggests its role in the production of adenosine triphosphate, a vital source of metabolic activity [16]. *M. hominis* utilizes arginine or hydrolyze urea for energy production, and this may increase ammonia levels as a byproduct in the blood. Unregulated ammonia level causes serious complications, such as hyperammonemia syndrome (HAS) resulting in brain edema and also necessitating continuous renal replacement therapy (CRRT) [17]. Therefore, at the onset of neurological changes, ammonia level should be tested in transplant patients positive for *M. hominis* [18].

*M. hominis* can be commensal and pathogenic. Upon invasion, *Mycoplasma* activates both innate and acquired immunity, eliciting acute and chronic inflammation. In turn, *Mycoplasmas* use unique mechanisms to escape immune response and colonize mucosal surfaces, invade different areas of the body [5, 19], and have the propensity to cause infection in transplant patients [5]. Notably, *Mycoplasmas* lack pathogen-associated molecular patterns (PAMPs) such as lipoteichoic acid, flagellin, and lipopolysaccharides. As a result, the host’s inflammatory response to the organism is independent of PAMPs. Additionally, the underlying mechanism of pathogenic determinants and the exact immune system evasion strategies employed by the organism are not fully characterized [5, 20]. The extragenital prevalence of *M. hominis* in transplant patients warrants genotypic profiling to understand the underlying mechanism of pathogenicity in solid organ transplant patients. In case 1, the donor was positive for *K. pneumoniae* but no diagnostics were performed to rule out *M*. *hominis.* The emergence of *M. hominis* infection 7 days post-transplant suggests donor-driven infection, as a small subset (1–3%) of healthy individuals carry *M. hominis* in the respiratory secretions [12], and the incubation time for the microorganism is two to seven days [21]. Conversely, in case 2, host immune status could be contributory as the patient developed the infection 614 days after the transplant. Divithotewala. C. et al., reported frequent *M. hominis* infections in transplant patients, and suggested that this increase may be due to infection prevalence in the general population [22]. Whether the surge in the incidence of *M. hominis* infection is a result of its prevalence in the population or a consequence of enhanced detection facilitated by the accessibility of sophisticated diagnostic tools, such as molecular techniques and specialized media, remains unclear.

*M. hominis* requires special media for optimal growth. The enriched medium, such as SP4 with arginine, is highly nutritious. The medium is supplemented with beef heart infusion, basal peptone with yeast extract, fetal bovine serum, sterols, and cholesterol. Amphotericin B and polymyxin B are added to the medium to inhibit rapidly growing contaminants and to promote the growth of *M. hominis*. SP4 agar and broth supplemented with arginine are incubated for 1–4 days in CO_2_ at 35 ^o^C and under aerobic conditions respectively. The colonies of *M. hominis* on the SP4 agar appear as tiny “fried-egg” or penetrating finely granular berry-like, while the broth transitions from original orange to red color [23, 24]. *Mycoplasmas* are susceptible to adverse environmental conditions. To detect *M. hominis*, specimens should be placed into appropriate transport or growth media, such as Mycoplasma transport broth or Mycoplasma/Ureaplasma transport media (UTM) like M4. The specimen remains stable for 8 h at ambient temperature and should be kept at 2–8 ^o^C if delay in testing is anticipated. Although SP4 and UTM media are ideal for the isolation and transport of the organism, special media are not routinely available in diagnostic laboratories [25–27]. Of note, *M. hominis* recovery from the blood culture bottle is hindered by the mycoplasmastatic effects of commonly used anticoagulant, sodium polyanethole sulfonate (SPS) [28]. Since the identification of the organism is crucial for prompt treatment, molecular techniques are expedient and can successfully identify the organism [29]. *Mycoplasmas* have been detected in body fluids using fluorescent stains, such as acridine orange and Hoechst 33,258. However, the application of these stains is limited due to their lack of specificity [30]. Thioglycolate (thio) broth is an enriched medium used for the recovery of fastidious and anaerobic organisms. Sodium thioglycolate in thio broth acts as a reducing agent and neutralizes the formed peroxides, and supports microorganisms’ growth [31, 32]. In our first case, thio broth, a routine microbiology medium, inoculated with the specimen led to the detection of *M. hominis*. While there are reports of extending the incubation period of standard media for the purpose of isolating *M. hominis* [33], it is crucial to consider the potential consequences associated with such an approach. This could result in delays in reporting the laboratory result and subsequent therapeutic intervention, thereby emphasizing the importance of careful consideration when using this methodology. MALDI-TOF MS effectively identifies *M. hominis* but necessitates colonies from solid media [34], which remains a challenge in diagnostic laboratories. Molecular techniques offer rapid means of identification, but their use is contingent upon their availability. Furthermore, for susceptibility testing, *M. hominis* culture remains irreplaceable [33]. To prevent therapeutic delays, *M. hominis* should be included on the list of pathogens to be ruled out in solid organ transplant patients.

Due to the absence of a cell wall in *M. hominis*, antibiotics such as β-lactams, and vancomycin are ineffective. *M. hominis* is also resistant to trimethoprim/sulfamethoxazole (folate antagonist) and aminoglycosides [35]. The susceptibility profile of *M. hominis* shows that it is inherently resistant to C-14 and C-15 membered macrolides, such as clarithromycin, erythromycin, and azithromycin, while sensitive to C-16 macrolide, josamycin [36]. In addition to doxycycline, which is the preferred treatment, fluoroquinolones, and clindamycin are also recommended for *M. hominis* treatment. However, these antibiotics are not included in the standard post-transplant prophylactic regimens [35, 37]. Importantly, susceptibility testing should be performed on the isolate, as the presence of the *tet*(M) gene, which encodes for a protein that mediates resistance by tetracycline ribosomal protection, can result in tetracycline resistance [38].

Herein, we summarized (Table 1) 10 studies of *M. hominis* infection in lung and heart transplant patients from December 2013-March 2023. Of the total 24 cases, 83% of the *M. hominis* infection was reported in males and 17% in females. Male patients’ age ranged from 21 to 70 years (M = 50.8, SD = 17.4), and females ranged from 18 to 65 years (M = 42.7, SD = 12.7). The mean duration of identification of *M. hominis* post-operative days ranged from 3 to 42 days (M = 20, SD = 12.3). In 75% of the cases, routine and special media were used for *M. hominis* isolation, 16.7% reported culture and molecular methods such as primer-specific PCR or 16 S rDNA sequencing or Real-time quantitative PCR (qPCR), and 8.3% used molecular techniques alone for the organism detection. Doxycycline, moxifloxacin, minocycline, azithromycin, clindamycin, and levofloxacin were among the treatment of choice [13, 22, 37, 39–45].

**Table 1:** Shows the summary of lung and heart transplant cases (2013–2023) and post-transplant *M. hominis* infection

Authors and year	Age (Years)	Sex	Underlying condition	Transplant	Complaint after transplant	Treatment	Method	POD (days)
Divithotewala, C., et al., 2023 ^(22)^	*	M	Congenital heart disease	LT	Sternal wound infection	Doxycycline azithromycin and moxifloxacin	Culture	28
Divithotewala, C., et al., 2023 ^(22)^	*	M	Cystic fibrosis	LT	Bilateral anastomoses ischemia, right anastomose dehiscence empyema	None (Pt expired)	Culture	28
Divithotewala, C., et al., 2023 ^(22)^	*	M	Chronic obstructive pulmonary disease	LT	BL anastomoses ischemia empyema	Doxycycline moxifloxacin	Culture	36
Divithotewala, C., et al., 2023 ^(22)^	*	M	Interstitial lung disease	LT	L’anastomose ischemia, sternal wound infection mediastinitis pericarditis	Doxycycline moxifloxacin	Culture	42
Vecchio, M., et al., 2021 ^(39)^	56	M	Rapidly progressive pulmonary fibrosis	BLT	Fever, hypoxemia, and pneumoniae	Doxycycline and oral moxifloxacin	Real-time quantitative PCR (qPCR)	9
Michel, C., et al., 2021^(37)^	55	M	Idiopathic pulmonary fibrosis	BLT	Acute respiratory distress	Azithromycin and doxycycline	Culture	12
Chang, S.Y., et al., 2021 ^(40)^	34	M	Congenital heart disease, cardiac cirrhosis	OHT, OLT	Pneumonia; PSI, pleural space infection, septic shock	Doxycycline Levofloxacin	Culture	14
Chang, S.Y., et al., 2021 ^(40)^	24	M	Systemic sclerosis-associated interstitial lung disease and pulmonary arterial hypertension	BLT	Pleural effusion	Doxycycline	Culture	11
Chang, S.Y., et al., 2021 ^(40)^	64	M	Chronic obstructive pulmonary disease	BLT	Altered mental status, septic shock, and hypoxemic respiratory failure	Doxycycline and levofloxacin	Culture	10
Chang, S.Y., et al., 2021 ^(40)^	63	M	Hypersensitivity pneumonitis	SL	Skin and soft tissue infection	Doxycycline, moxifloxacin	Culture	37
Chang, S.Y., et al., 2021 ^(40)^	68	M	Idiopathic pulmonary fibrosis	SL	Extensive hemorrhage, hypoxemic	Levofloxacin and doxycycline	Culture	29
Chang, S.Y., et al., 2021 ^(40)^	31	M	Idiopathic pulmonary fibrosis	BLT	Persistent fevers and a unilateral pleural effusion	Levofloxacin and doxycycline	Culture	11
Chang, S.Y., et al., 2021 ^(40)^	43	M	Hypersensitivity pneumonitis	BLT	Septic shock and hypoxemic respiratory failure	Doxycycline and levofloxacin	Culture	9
Givone, F., et al., 2020 ^(41)^	28	M	Thoracic aortic aneurysm associated with aortic insufficiency	HT	High-grade fever, an aortic murmur without signs of heart failure	Moxifloxacin	16 S ribosomal DNA PCR	NS
Dixit, A., et al., 2017 ^(13)^	18	F	Monosomy 7 and Myelodysplasia	BLT	Chronic bronchitis with a mixed inflammatory infiltrate and variable mural fibrosis	Moxifloxacin and clindamycin	Culture	20
Hagiya, H., et al., 2017 ^(42)^	21	M	Restrictive cardiomyopathy and pulmonary hypertension	HLT	Respiratory failure	Minocycline and levofloxacin	Culture	36
Smibert, O.C., et al., 2017 ^(43)^	46	F	Bronchiolitis obliterans secondary to seropositive rheumatoid arthritis	BLT	Progressive respiratory failure, bilateral interstitial infiltrates, and pleural effusions	Moxifloxacin and doxycycline	Culture	19
Smibert, O.C., et al., 2017 ^(43)^	65	M	Non–cystic fibrosis-related bronchiectasis	BLT	Worsening respiratory function	Moxifloxacin	Culture	3
Smibert, O.C., et al., 2017 ^(43)^	51	M	Cystic fibrosis-related bronchiectasis	BLT	Right hip and groin pain	Moxifloxacin and doxycycline	Culture	29
Sampath, R., et al., 2017 ^(44)^	63	M	Idiopathic pulmonary fibrosis	BLT	Right bronchial anastomotic leak	Levofloxacin and doxycycline	PCR and culture	NS
Sampath, R., et al., 2017 ^(44)^	70	M	Idiopathic pulmonary fibrosis	SL	Pleuritis	Doxycycline	PCR and culture	NS
Sampath, R., et al., ^(44)^	64	M	Chronic obstructive lung disease	SL	Pleuritis	Doxycycline	PCR and culture	NS
Sampath, R., et al., 2017 ^(44)^	64	M	Idiopathic pulmonary fibrosis	SL	Pleuritis	Levofloxacin and doxycycline	PCR and culture	NS
Wylam, M.E., et al.,2013 ^(45)^	64	F	Pulmonary fibrosis	BLT	Severe encephalopathy	None (Pt expired)	Culture	4

POD = Postoperative days, NS = Not specified, BLT = Bilateral Lung Transplant, SL = Single Lung Transplant, HT = Heart Transplant, HLT = Heart Lung Transplant, LT = Lung Transplant, OHT = Orthotopic Heart Transplantation, OLT = Orthotopic Liver Transplantation

(* mean age reported 56 years), None (patient expired)

## Summary

*M. hominis* is a fastidious commensal organism of the genital tract. *M. hominis* lacks a cell wall, which poses a challenge in detecting the organism using routine diagnostic microbiology methods. We present a rare occurrence of extra-urogenital *M. hominis* infection in two transplant patients. A high incidence of morbidity associated with *M. hominis* infection in solid organ transplant patients warrants clinicians to be aware of the laboratory limitations and submit the appropriate requests to be sent to reference laboratories if necessary.

## Data Availability

All data generated or analyzed during this study are included in this published article.
